# United States Veterinary Medical Association

**Published:** 1893-10

**Authors:** 


### UNITED STATES VETERINARY MEDICAL ASSOCIATION.

One year ago, in Boston, at the twenty-ninth annual meeting of the United States Veterinary Medical Association, a considerable amount of enthusiasm was aroused by the resolutions and discussions which planned for and considered the ways and means which would be adopted to prepare for the thirtieth annual meeting of the Association, and the first Veterinary Congress of America. Chicago, the seat of the World's Fair, was chosen, for many reasons, as it has been by the numerous other congresses which have met in America this year. How judicious this choice has been, we will be better able to know in another month, when the meeting has been held and is one of our records of the past. The committees were appointed with care by the officers of the Association, following the laws of specialization, and fitting into each section and department of work such members as seemed adapted to the work required by the demands of all. Hearty co-operation was expected of each individual and association of individuals on the committees, and undoubtedly each and all formed the best of resolutions to do his utmost to deserve the trust imposed, and to put aside part of his own personal affairs, in order to contribute to the success of the professional organization. But good resolutions alone will not pave the way smoothly, even to Chicago. Many a member, when he left the hospitable boards of our Boston meeting, and returned from the stimulating intercourse with his colleagues to the routine of pneumonia and greasy-heels, colic and corns, and feed bills and collections, forgot and neglected the æsthetic part of his professional duty, and the affairs of the Association fell into the position of the hedge-hog which was the mutual property of two boys: both boys saw him; one got his fingers bitten catching him, and then labored daily feeding him until show time came, when the other boy appeared and claimed his share of partnership in the possession of the animal. I very much fear that many of us will go to Chicago most shamelessly, and with pride claim partnership in all that is good in the Association, while we have left all care and labor in feeding and nursing the beast to our diligent and efficient Secretary, Dr. Hoskins, and a few others who have aided him.

The following reports of committee meetings and circulars are an outline of what has been done for our benefit :

JANUARY 1, 1893.

*Notice to all Members United States Veterinary Medical Association:* Upon recommendation of the Comitia Minora, the following resolution, approved by the Association, carrying with it the power of levying an assessment, was adopted at Boston, September 20, 1892:

*Resolved*, That a special assessment of $5.00 be levied on each member to defray the extraordinary expenses that will be incurred by the International Meeting at Chicago in 1893.

Said assessment is now due and may be sent to the Secretary's office by check, money order or draft.

W. HORACE HOSKINS, Secretary.

W. L. WILLIAMS, President.

---

*Notice to Members United States Veterinary Medical Association:* I have the pleasure of announcing to the members of the Association, and the profession in general at home and abroad, that there will be two valuable contributions offered to our Association at its International Meeting, which should attract the attention and interest of the entire profession of the world. They are the result of several years' investigation on the subject of "Swine Plague" and "Hog Cholera" and "Contagious Pleuro-Pneumonia."

The first paper will be offered jointly by Dr. A. W. Clement and Dr. William Welch. The second paper will be offered by Dr. A. W. Clement, and it will be accompanied by the most exhaustive collection of pathological specimens which have ever been gathered together in the world.

W. HORACE HOSKINS, Secretary.

---

*To the Members of the U. S. Veterinary Medical Association and all Members of the Veterinary Profession in the United States and throughout the world:* The United States Veterinary Medical Association has selected the dates of October 17th, 18th, 19th and 20th for their International Congress, to be held on the grounds of the World's Fair at Chicago, Ill., in conjunction with the World's Fair Auxiliary Congresses; and all National, State and local associations throughout the world are hereby extended a most cordial invitation to send delegates to the Congress, and to take part in the deliberations of the same.

Every arrangement that is possible will be made in the interest of all those who desire to attend, and information as to railroad rates, hotel accommodations, and all other conveniences will be cheerfully afforded by addressing the Secretary of the Association.

W. HORACE HOSKINS, Secretary,
12 South 37th St., Philadelphia, Pa.

By order of the President,
W. L. WILLIAMS.

The Committee on Prizes of the United States Veterinary Medical Association desires to call the attention of its members to the following announcement: It is the purpose of the Association to give the sum of $50, to which the editors of the *American Veterinary Review* and JOURNAL OF COMPARATIVE MEDICINE have each added $25, as a first prize for the best paper that may be submitted to them on any professional subject.

In addition to this the editors of the *American Veterinary Review* and the JOURNAL of COMPARATIVE MEDICINE will each give plate to the value of $25, making a total prize of $50 for the second best paper, as above. Paper for this competition must be legibly written and in the hands of the committee by August 1, next.

In compliance with an order of the Association, the prize paper must be read and defended at one of the regular meetings. The successful paper in this competition will probably be called up at the coming annual meeting in Chicago, at which time and place the prizes will be awarded, the final award being made by the committee.

The article is then to become the property of the Association.

Competitors shall use a *nom de plume*, retaining in their own way the means of after identification.

Paper should be addressed to Charles P. Lyman, chairman, 50 Village Street, Boston, Mass.

C. P. LYMAN, Chairman, 50 Village St., Boston,<br>

W. H. LOWE, 190 Ellison St., Paterson, N. J.,<br>

L. MCLEAN, 14-16 Nevins St., Brooklyn, N. Y.,<br>

} Committee.

---

*To the Members of the United States Veterinary Medical Association and the Profession in general:* I have the pleasure of announcing that Prof. Olaf Schwartzkopf, of St. Paul, Minnesota, will offer a paper at the International Veterinary Congress in Chicago, in October next, entitled "Comparative Psychology of our Domestic Animals." A paper the outcome of studies, investigations and close attention given by the author, and his recognized ability as a writer and teacher makes the promise of this paper one of exceptional merit.

The reprints and papers of the last two meetings of the United States Veterinary Medical Association at Washington and Boston will be ready for delivery about the 20th of May. All those who are not members of the Association but who are desirous of receiving the same may obtain them through the secretary at the cost of their publication.

The new application blanks are now ready, and may be obtained from any of the assistant state secretaries or from the office of the general secretary upon application. All applications for membership must be filed in the office of the secretary on or before the first of October next, in order to be insured consideration at the Chicago meeting.

By order of the President.

W. L. Williams,
Lafayette, Ind.

W. Horace Hoskins, Secretary,
12 South 37th Street,
Philadelphia, Pa.

---

Philadelphia, May 25, 1893.

*To the Members of the United States Veterinary Medical Association:*

The reprints of our proceedings, containing a complete account of our meetings at Washington and Boston for 1891-2, will emerge from the printer's hands in the next ten days.

These reprints will contain all the papers, reports of committees; discussion; with a list of officers, past and present; those who have resigned; the deceased members; those dropped from the rolls; those expelled, and much other information relative to the Association that it will be found of interest and value as a book of reference.

This book will be bound in cloth, with gilt top, for 25 cents, while the cost bound in paper will be but 5 cents. All those desiring that their copies shall be bound in gilt will immediately give notice to this office, and transmit 25 cents by postal note or postage stamps. Copies of these reprints will be issued to all members of the Association. To all others desiring a copy the exact cost of printing and binding will be charged for the same.

I desire to give notice at this time of the near approach of our International Congress at Chicago, and to say to the members that every one has a duty to perform in making this meeting a grand success. The officers and committees are actively at work, and the three leading subjects that are to be considered and discussed will be handled in a very thorough and interesting manner. The general committee reports will be of special interest, and in addition we have offered the following papers:

"Swine Plague and Hog Cholera,"—Drs. Welch and Clement.

"Contagious Pleuro-Pneumonia,"—Dr. A. W. Clement.

"Millet Disease of Horses,"—Dr. T. D. Hinebaugh.

"Fistulæ,"—Dr. M. H. Reynolds.

The consideration of our Association having a National charter under the United States Government will be acted upon at this meeting.

A list of honorary members will be considered that has for its aim a proper recognition of veterinarians in every country of the world who have contributed largely to the general advancement of the profession.

Many other matters of equal importance will be taken up and disposed of at this meeting, and it is urged that every member of the Association shall lay aside his business and work and attend this coming meeting, as it will probably be the only International Congress ever held in America during the lives of the present membership of our Association ; and its importance and value will wholly depend upon the united effort of those upon whom the responsibility of its success depends, and this is upon our members.

From time to time bulletins will be issued from the secretary's office, containing details of plans and arrangements, railroad rates, etc., in ample time for the proper preparation of our meeting, and at a time when the best rates may be obtained.

By order of the President.

W. L. WILLIAMS, President.

Lafayette, Ind.

W. HORACE HOSKINS, Secretary,
3452-54 Ludlow St., Phila., Pa.

---

*To the Members of the United States Veterinary Medical Association:* The Comitia Minora of this Association will convene in Chicago on October 16th for the discussion of all preliminary business and the consideration of all applicants for membership.

The officers of the Association will arrange for headquarters at one of the leading hotels, where they may be reached at certain hours by all those who desire information or assistance of any kind.

The Illinois Association will tender to the members of the Congress an evening entertainment on the lakeside, with a lunch, music, etc.

The members of the Local Committee of Arrangements in Chicago will place at the disposal of the members of the Congress one or more of their members during our stay in Chicago, to guide them to any place of amusement and point of interest in the city or the surroundings.

The resignation of Dr. M. Stalker on the Committee of Veterinary Education has been accepted, and Dr. Niles, of Ames, Iowa, has been appointed in his place.

The officers have accepted a paper from Dr. T. D. Hinebauch, of Fargo, North Dakota, entitled "Millet Disease of Horses."

The local arrangements are rapidly being completed, and the Secretary will be glad to furnish information at any time, to any member of the profession, by forwarding their requests to his office.

W. HORACE HOSKINS, Secretary.

Philadelphia, Pa.

By direction of the President,
W. L. WILLIAMS,
Lafayette, Ind.

---

U. S. VETERINARY MEDICAL ASSOCIATION.
SECRETARY'S OFFICE.

PHILADELPHIA, July 1, 1893.

*To the Members of the Veterinary Profession:*

The First International Veterinary Congress to be held in America, will convene at the World's Fair, Chicago, Ill., October 16th, and will continue until the 20th, inclusive.

The special subjects to be considered at this Congress are: Veterinary Education, Tuberculosis, and Animal Food Supply.

Veterinarians from all parts of the world are cordially invited to attend, present papers and take part in the discussion.

All veterinarians in good standing may become members of this Congress on the payment of two dollars, which will entitle them to a copy of the proceedings.

All foreign Veterinary Colleges, Schools and Societies are requested to appoint delegates.

Papers on all subjects of interest to the veterinary profession are cordially solicited.

In order to receive proper assignment on the program, papers should be filed in the office of the Secretary at least two weeks before the opening of the Congress, together with the author's name and the title of his paper.

For further information address the Secretary,

W. HORACE HOSKINS, 3452–54 Ludlow Street,
Philadelphia, Pa

INTERNATIONAL COMMITTEE.

R. S. Huidekoper, 155 West 56th St., N. Y. City.
A. Liautard, 141 West 54th St., N. Y. City.

D. E. Salmon, Bureau of Animal Industry, Washington, D. C.
A. H. Baker, 145 Michigan Avenue, Chicago, Ill.
J. H. Stickney, American Stables, Sudbury St., Boston, Mass.
Olof Schwartzkopff, 637 Cedar St., St. Paul, Minn.

EX-OFFICIO.

W. L. Williams, Bloomington, Ill.
A. W. Clement, 902 Cathedral St., Baltimore, Md.
W. Horace Hoskins, 3452–54 Ludlow St., Philadelphia, Pa.
J. L. Robertson, 409 Ninth Ave., N. Y. City.

---

### MEETING OF THE COMITIA MINORA, INTERNATIONAL COMMITTEE, AND OTHER COMMITTEES OF THE UNITED STATES VETERINARY MEDICAL ASSOCIATION.

The meeting was held in the office of Dr. Huidekoper, at 129 West 52d St., New York, May 20th, 1893, and was attended by the following members: A. W. Clement, of Baltimore, Vice-President; James L. Robertson, of New York, Treasurer; W. Horace Hoskins, Secretary; Leonard Pearson, Philadelphia, A. Liautard, New York. R. A. McLean, of Brooklyn, as a guest.

The Secretary called for report of the International Committee, who asked a delay of half an hour for preparation of same. The Secretary then read the following communication from the President, Dr. Williams:

PURDUE UNIVERSITY,
LAFAYETTE, IND., May 17th, '93.

*Dr. W. Horace Hoskins, Sec'y U. S. V. M. A.*

DEAR DOCTOR:—Your notice of May 12th of meeting of committees of the United States Veterinary Medical Association at New York City on the 20th inst., to complete arrangements for International Meeting, duly at hand and noted.

I regret very much that my duties here forbid my attending, but I trust Dr. Clement will be present to act in my stead, and will cheerfully abide by the action taken by the International Committee or the Comitia Minora. I have little to suggest that is new.

The local arrangements at Chicago will be all right, and I shall be in Chicago within four to six weeks and complete the details as far as practicable at that date.

The dates to be assigned us for public mass meeting have not yet been decided upon, neither have the speakers, nor any one of them for this part.

Of these we should have five or six addresses twenty or thirty minutes long, and I suggest that the speakers be largely from foreign countries, possibly two from the United States and Canada, the others from England and continental Europe. All must, of course, be able to speak English, and make their addresses in our language.

I think Professors McEachran and Smith should be provided with or offered places on programme. Can they not have places on Veterinary Education?

As subjects for addresses in mass meeting I would respectfully suggest:

1. History of Veterinary Science.
2. Veterinary Science and National Economics.
3. Veterinary Science and National Health.
4. Veterinary Science in Relation to Ethics.
5.
6.

Will leave some blanks; possibly those already suggested will be deemed unsatisfactory.

These topics should be settled at once, and proper persons for presenting them named, with alternates where practicable.

The Committee on Honorary members will doubtless submit a satisfactory report, and in making list of foreign veterinary associations and colleges I have little to suggest or knowledge to impart, but let me suggest for one thing that we try if possible to have representatives from Central and South America.

I have accepted a paper from Dr. M. H. Reynolds, Keosauga, Iowa, on "Fistulæ" which comprises considerable work, and promises to be interesting.

Permit me to suggest that the matter of programme, its arrangement, etc., be well discussed, suggestions made as to general outline, and the details then committed to the judgment of a small committee to carry out the recommendations as far as practicable.

We can have all the hall room we wish, and in case the major subjects already fixed upon by the Association will require the full time of the meeting, I suggest that subordinate, or rather branch meetings be held for the consideration of other topics, to allow veterinarians to choose between two programmes, and attend the one of most interest. Some veterinarians may not care to hear discussions on our major subjects. The details of such arrangements I would leave with Programme Committee. I have no further papers under consideration.

Any appointments which need immediate attention please refer to Dr. Clement, or in case the appointment should come from the International Committee, then, of course, to Dr. Huidekoper.

This is all which now occurs to me, and should I have anything to add, will address you at New York, care of Dr. Liautard, either by telegraph or post.

Have written Mr. Bonney on several matters which I trust will be answered in time to lay the information before the meeting.

Wishing a successful meeting, and regretting my inability to be with you in person, I am

Very truly yours,

W. L. Williams.

The Secretary then read a letter from Dr. C. P. Lyman, of Boston.

Harvard University,
Boston, Mass., May 15th, '93.

*W. H. Hoskins, D. V. S.,*
*12 South 37th St.,*

My Dear Doctor:—Your favor of May 11th is received. I am sorry to say that I shall not be able to be present in New York on the 20th inst. As being the one appointed by the Comitia Minora at their last meeting in New York to see what could be done toward obtaining a national charter for our Association, I have to report the receipt of the following letter from Washington, dated February 11th, 1893, and signed by one of the United States Senators:

"It is now too late in the session to do anything with any new bill in this Congress, but if you have an act of incorporation drawn I think there will be no difficulty in getting it through Congress at next session. These bills are usually passed without objection. Any good lawyer could draw it for you."

I should like to be further instructed by the committee as to whether I shall employ a lawyer to draw an act of incorporation for us as suggested by the Senator. There seems to be some good reason for thinking that Congress will be called early in the fall, and if we are to do anything it would seem to me that we had better be prepared to advance our request there at a very early date.

As Chairman of the Committee on prizes, I have to state that considerable work has been done, as a result of which, and through the kindness of the editors, we have been able to publish in the professional journals a notice asking that papers be submitted to the committee.

I am happy to be able to say that the form of this notice has finally assumed a shape that seems to be fairly acceptable to all those interested.

No papers have as yet been received.

Will you kindly bring these matters before the committee for me, and advise me of their further desires.

Yours very truly,

CHARLES P. LYMAN.

A letter from Dr. J. F. Winchester, Lawrence, Mass., was also read.

LAWRENCE, MASS., May 14th, '93.

*Dr. W. Horace Hoskins.*

DEAR DOCTOR:—Yours of the 12th at hand. It will be impossible for me to come to New York on the 20th. As to the work being done by the committee of which I am Chairman, I would say that Dr. Wyatt Johnson has promised an article on Cornstalk Disease and Contagious Pleuro-Pneumonia of the English. Dr. J. B. Paige will take up the subject of Tuberculosis. Dr. S. Stewart will attend to the Swine. Dr. Jos. Hughes has not even shown me the courtesy or good breeding to reply to either of two letters I have sent him suggesting that he take the subject of Actinomycosis. I will try in a general way to fill up the report, that it may be acceptable for the occasion.

Yours, etc.,

J. F. WINCHESTER.

The Secretary then read the list of those who were invited to report. They were the International Committee, Comitia Minora, Committee on Veterinary Education, Animal Food Committee and Committee on Tuberculosis.

The Secretary then asked that the names of the candidates for honorary membership be produced. This, however, could not be done, as the committee who had charge of making out the list had been too busy to complete it.

Dr. McLean asked whether persons who were honorary members were included among those whose expenses had to be paid.

The Chairman replied that it had already been decided not to pay their expenses.

Dr. Liautard moved that the committee be empowered to have a letter of invitation and circular, explaining the object of the Congress, written in English, French and German, and containing the names of those recommended by the committee for honorary membership, and that these letters be sent to the veterinary papers of the various countries with the request that publicity be given them. This motion was seconded and passed.

In reply to questions asked by some of the members, the Secretary stated that it had been resolved that three persons from each of the various countries should be the maximum number for honorary membership, and that these were to be selected by the committee for approval by the Association. He said also that if publicity were given to the names of those who had been selected as candidates for honorary membership, it would be an incentive for them to come to our meeting.

He said that it had been decided at the Boston meeting that any member could submit to the committee the name of any one whom he deemed worthy of membership. The names of worthy candidates should be put in print by July at the latest.

The Committee on Honorary Membership reported progress.

Dr. Huidekoper then read the invitation which he had formulated. It was considered sufficiently comprehensive, and needed only further development and greater detail.

The International Committee reported progress, and requested that they be given six days to complete their circular and be empowered to publish it.

The Secretary reported that the Pennsylvania Association stand ready to respond to any call that may be made by the officers in charge of the Congress, and that they will do everything in their power for the promotion of its success. They have appointed a committee for considering any work that may be designated by this Association, and they have also appointed a committee for the entertainment of foreign delegates on their way to Chicago.

The Secretary then called the attention of the Comitia Minora to the reapplication of John Doris, Jr. The latter was a graduate of the Cincinnati Veterinary College, and was rejected on the ground that he was a graduate of a school granting diplomas to minors. The school has now changed, and does not confer diplomas on those who are under twenty-one years of age. The Dean of the Faculty has written for the exact wording of the rejection of Dr. Doris, who will reapply for membership.

Adjourned to meet at the call of the Secretary.

The following program has been issued: 30th Annual Meeting of the United States Veterinary Medical Association and the first Veterinary Congress of America, World's Fair Memorial Art Palace, Michigan Avenue, foot of Adams Street, Chicago, Illinois, October 16, 17, 18, 19 and 20, '93.

*International Committee*—W. L. Williams, President, Bloomington, Ill.; A. W. Clement, Vice-President, 902 Cathedral St., Baltimore. Md.; W. Horace Hoskins, Secretary, 3452-54 Ludlow St., Philadelphia, Pa.; J. L. Robertson, Treasurer, 409 9th Ave., New York City; R. S. Huidekoper, Chairman, 155 W. 56th St., New York City; D. E. Salmon, Bureau of Animal Industry, Washington, D. C.; A. H. Baker, 145 Michigan Ave., Chicago, Ill.; J H. Stickney, American Stables, Sudbury St., Boston, Mass.; Olaf Schwarzkopf, 445 Roberts St., St. Paul, Minn.

*Comitia Minora*—W. L. Williams, President, Bloomington, Ill; A. W. Clement, Vice-President, 902 Cathedral St., Baltimore, Md.; W. Horace Hoskins, Secretary, 3452-54 Ludlow St., Philadelphia, Pa.; J. L. Robertson, Treasurer, 409 9th Ave., New York City; R. S. Huidekoper, 155 W. 56th St., New York City; A. Liautard, 141 W. 54th St., New York City; R. A. McLean. 14 Nevins St., Brooklyn, N. Y.; S. Stewart, Nebraska City Neb.; T. J. Turner, Columbia, Mo.; C. E. Hollingsworth, La Salle, Ill.; J. F. Winchester, Lawrence, Mass.

*Local Committee of Arrangements*—S. S. Baker, Chairman, 901 Jackson Boulevard, Chicago, Ill.; R. J. Withers, 2537 State St., Chicago, Ill.; G. W. Pope, 2537 State St., Chicago, Ill.

PROGRAM.

*Monday, October 16, 1893.—Special Committee Meetings.*—10 A. M., Special Meeting of Local Committee of Arrangements. 11 A. M., Special Meeting of International Committee. 1 P. M., Special Meeting of the Comitia Minora.

*Tuesday, October 17, 1893.*—8.30 A. M., Regular Meeting of the Comitia Minora. 10 A. M., Annual Meeting convened.

*Roll Call.*—Address of welcome to the members by President W. L. Williams, Bloomington, Ill. Address of welcome to the visitors. Reception and consideration of report of Comitia Minora. Report of Committee on Honorary Members.

*Unfinished Business.*—1 P. M., Adjournment for lunch. 2 P. M., Reconvened.

*Reports of Committees.*—Special Committee on International Meeting; Committee on Intelligence and Education; Finance

Committee; Committee on Diseases; Prize Committee; Committee on Army Legislation; Publication Committee; Special Committee on Incorporation; Secretary's report; Reports from Assistant (State and Foreign) Secretaries.

*Reading of Papers.*—"Millet Disease of Horses," Dr. T. D. Hinebauch, Fargo, North Dakota; "Biliary Hepatitis in Cattle" (Pictou Cattle Disease), Dr Wyatt Johnston, Montreal, Canada.

*Wednesday, October 18, 1893*—9 A. M. to 1 P. M., popular meeting under the direction of the World's Fair Auxiliary Congress. "Horseshoeing," Drs. Pearson and Huidekoper; "Psychology of Domestic Animals"—Part 1st, Historical Introduction; part 2d, Scientific part; part 3d, the Conclusion and Theses, Dr. Oloff Schwartzkopf, St. Paul, Minn. "Genus Hymoenlepis in Rats," Dr. S. E. Weber, Lancaster, Pa. "Has Contagious Pleuro-Pneumonia been entirely eradicated from the United States," Dr. J. W. Gadsden, Philadelphia, Pa. 1 P. M., Adjournment for lunch. 2 P. M., Discussion of reports of committees.

*Reading of Papers.*—"Fistulæ," Dr. M. H. Reynolds, Keosauqua, Iowa; "New Method of Treating Periodic Ophthalmia by Surgical Interference," Dr. R. H. Harrison, Atchison, Kansas. Election of Officers. New Business. 8 P. M., Reception by the Western Veterinarians.

*Thursday, October 19, 1893.*—Special topic for consideration. 10 A. M., "Veterinary Education," Dr. A. Liautard, Chairman. This will be considered under the following head: 1st, Veterinary education as it was; 2d, Veterinary education as it is; 3d, Veterinary education as it ought to be. Discussion of Veterinary education. 1 P. M., Adjournment for lunch. 2 P. M., Reconvened.

*Reading of Papers.*—"Swine Plague and Hog Cholera," Drs. W. H. Welch and A. W. Clement, Baltimore, Md. 6 P. M., Adjourn.

*Friday, October 20, 1893.*—Special topic for consideration; 10 A. M., "Animal Food," Dr. E. E. Salmon, Chairman; 1st, The measures necessary to keep meat-producing animals free from dangerous diseases prior to marketing; 2d, Meat inspection from the standpoint of public health; 3d, Meat inspection from the standpoint of national economy. Discussion on animal food. 1 P. M., Adjournment for lunch. 2 P. M., Reconvened; Tuberculosis Committee, Dr. A. W. Clement, Chairman; 1st, Nature and extent of the disease; 2d, Means of controlling the disease; 3d, Practicability of its eradication from our meat and milk producing animals. Discussion of Tuberculosis. Discussion of papers and reports of cases. 7 P. M., Banquet.

The programme which has been arranged by the officers of the Association has but one fault, and that is that it offers so much of interest and so many valuable subjects that time can scarcely be expected to be found in which to receive them, and still less to consider them as they deserve. But this is more than counterbalanced by the creditable labor which the program shows has been done, and the other original work which will be presented can soon be placed in type and studied at leisure later by one's own fireside, where it can be digested, and the good and the bad be separated for approval and criticism, in further writings and discussions at future meetings. Each and all, the delinquents most of all, owe their support to and courteous recognition of the works of those who have been faithful to the interests of all.

